# Different measles outbreaks in Belgium, January to June 2016 – a challenge for public health

**DOI:** 10.2807/1560-7917.ES.2016.21.32.30313

**Published:** 2016-08-11

**Authors:** Tine Grammens, Virginie Maes, Veronik Hutse, Valeska Laisnez, Carole Schirvel, Jean Marie Trémérie, Martine Sabbe

**Affiliations:** 1 Service of Epidemiology of Infectious Diseases, Department of Public Health and Surveillance, Scientific Institute of Public Health, Brussels, Belgium; 2 These authors contributed equally to this article and share first authorship; 3 National Reference Centre for measles, mumps and rubella, Service of Viral Diseases, Scientific Institute of Public Health, Brussels, Belgium; 4 Infectious Disease Control and Vaccination, Flemish Agency for Care and Health, Brussels, Belgium; 5 Infectious Disease Surveillance Unit, Agence pour une Vie de Qualité (AVIQ), Région Wallonne, Charleroi, Belgium; 6 Health Inspection (Service de l'Inspection de l'Hygiène de la Commission communautaire commune), Brussels, Belgium

**Keywords:** Measles, epidemiology, outbreak, vaccine-preventable diseases, elimination

## Abstract

During the first half of 2016, several outbreaks of measles were reported in the three regions of Belgium. Main challenges for public health were severe complications occurring in adults, nosocomial transmission and infection in healthcare workers. Here, we describe those outbreaks and lessons learnt for public health.

Measles has not yet been eliminated in Belgium according to the Regional Verification Commission for measles and rubella elimination in Europe [1]. Since the last large outbreak in 2011 [2] with an estimated incidence of 54.9 per 1 million person-years, measles incidence varied from 3.5 to 6.1 per 1 million person-years between 2013 and 2015 [3]. Here, we describe several small measles outbreaks occurring during the first half of 2016, based on preliminary data collected up to 30 June 2016.

## Definitions and reporting

The case definition of the European Union (EU) Commission Decision of 2012 was used and cases were classified as possible, probable or confirmed depending on clinical criteria, epidemiological link and laboratory criteria as described [4]. This case definition has been adopted by the regional health authorities in Belgium for standard reporting of measles.

A measles outbreak was defined as two or more laboratory-confirmed cases which are temporally related (with dates of rash onset occurring between 7 and 18 days apart) and epidemiologically and/or virologically linked [5].

Measles cases are under mandatory reporting to the regional health authorities, in charge of the epidemiological investigation and control measures [6,7]. Notifications from the regional health authorities and results from the National Reference Laboratory for measles are collected and analysed at the Belgian Scientific Institute of Public Health.

## Outbreak description

**Figure 1:**
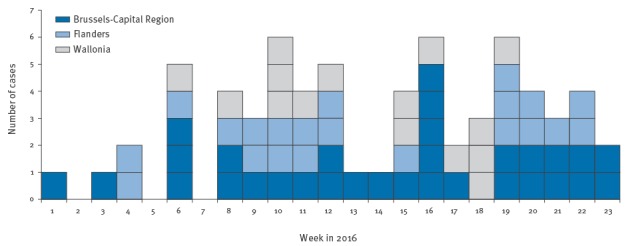
Reported measles cases by week of disease onset, Belgium, January–June 2016 (n = 67)

From the beginning of 2016 until 30 June, 10 measles outbreaks involving two to nine persons and 24 isolated cases have been identified in the three regions of Belgium, resulting in a total of 67 cases. For the 24 isolated cases, no epidemiological link was found, but we included them here based on the assumption that they had unknown links with the 10 outbreaks, given the time and place of occurrence of the large majority of cases. The last measles case was reported on 14 June 2016 (Figure 1). 

**Figure 2:**
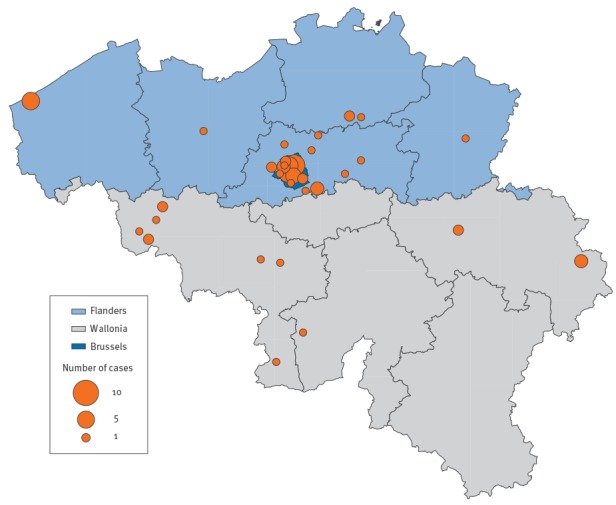
Geographical distribution of measles cases by province, Belgium, January–June 2016 (n = 67)

There were 31 cases in the Brussels Capital Region, 21 in Flanders and 15 in Wallonia (Figure 2). Incidence in Belgium (for the period January to June) was 6.0 per million. Incidence by region for the same period was 26.2 per million for Brussels, 4.2 per million for Wallonia and 3.3 per million for Flanders. For six cases in Flanders and two cases in Wallonia, an epidemiological link with the outbreaks in Brussels was described. 

Different transmission routes were identified among the 67 cases: household (12 cases), nosocomial (14 cases) and other (four cases); for the remaining 37 cases, the path of transmission was unknown. Four healthcare workers were infected, of whom three were unvaccinated and one had unknown vaccination status. Moreover, two cases had travelled to Romania, one to Poland and one to the United Kingdom (UK), all within the incubation period. Measles outbreaks were ongoing in these countries during their visits; however, a virological link has not yet been found for Romania and Poland. For the UK, a possible virological link is described in the chapter on laboratory confirmation. Three cases belonged to the Roma population. Four cases occurred in an asylum centre.

## Characteristics of the cases

**Figure 3:**
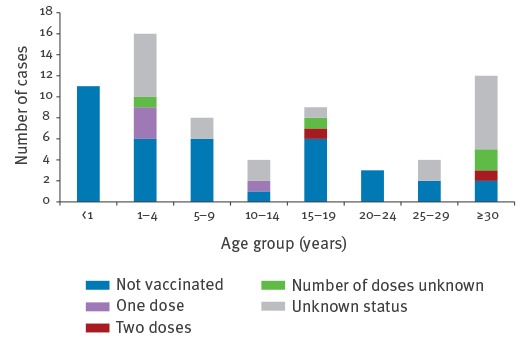
Age group and vaccination status of reported measles cases in Belgium, January–June 2016 (n = 67)

Of all cases, 27 were younger than five years, 12 were between five and 14 years-old, nine were between 15 and 19 years-old and 19 were older than 19 years (Figure 3). Two cases were vaccinated with two doses, four cases with one dose, four cases with an unknown number of doses, 37 cases (26 when excluding those younger than one year) were not vaccinated, and for 20 cases, of whom nine were older than 25 years, the vaccination status was unknown. Reasons for non-vaccination were, besides age below one year (11 cases), more frequently related to illness, hesitancy or previous side effects of the vaccine than to distrust or anti-vaccine beliefs.

Overall, 28 cases were hospitalised. The majority of hospitalised cases were children younger than five years (12 cases), children between five and nine years-old (four cases) and adults older than 25 years (eight cases). All hospitalised cases recovered. Among the children, only one was admitted with severe complications. Among the adults, three presented with severe complications: two with rhabdomyolysis with need of intensive care and one with hepatic cytolysis. No deaths were reported.

## Laboratory confirmation

Overall, 53 of the 67 cases were laboratory-confirmed by detecting measles virus-specific IgM antibodies and/or viral RNA by RT-PCR. Another eight cases were confirmed by an epidemiological link with a confirmed measles case. The National Reference Centre (World Health Organization (WHO)-accredited) confirmed 38 cases. For the remaining 15 cases, samples were confirmed by proficient (BELAC-accredited) laboratories but not sent to the National Reference Centre. Genotyping was done for 33 cases and genotypes D8 (two cases) and B3 (31 cases) were detected. Genotype D8 was found in a cluster of two persons; the index case had stayed in the UK during the incubation period. For genotype B3, different subtypes were confirmed by the National Reference Centre, namely MVs/Allada.BEN/3.10 (eight cases) and MVs/Tonbridge.GBR/5.14 (23 cases). The sequences of the isolates were analysed using the MeaNS database [8], where each sequence was entered to determine the genotype and to look for an identical sequence/match. This database gave us the opportunity to look for sequences/genotypes circulating in the neighbouring countries. The MVs/Allada.BEN/3.10 strain was found in Flanders and Wallonia and was related to strains found in outbreaks in France (Calais), Italy, Romania and the UK. The MVs/Tonbridge.GBR/5.14 strain, mainly found in Brussels but also appearing in the neighbouring provinces of Flanders and Wallonia was related to strains found in Villeneuve St George, France in 2016. 

## Control measures

Regional public health authorities took control measures according to their guidelines [6,7]. Control measures included thorough source investigation, ring vaccination and contact tracing by telephone to inform contacts and take preventive measures. Persons that had contact with a case less than seventy-two hours before and who were not immune or did not know their immune status were vaccinated. Persons were considered immune if they had received two vaccines, if they had had measles in the past or if they were born before 1970 [6,7]. If the contact was a child between six months- and one year-old, they were also vaccinated. In these cases, the child still has to receive two vaccines after the age of one year to be immune [6,7].

Because of several nosocomial infections and adults with severe clinical presentations, a consultative risk assessment with the health authorities, including those of the three regions, was held on 14 April 2016. Following this assessment, letters were sent to hospitals and general practitioners of the most affected areas. In addition, the Superior Health Council was asked for scientific advice on issuing a specific recommendation for vaccination of risk groups (healthcare workers and persons working with children). Healthcare workers in Belgium are not required to show evidence of MMR vaccination in healthcare settings. Moreover, systematic measles vaccination was also offered to all asylum seekers.

During the European vaccination week (25–30 April 2016), an information campaign in Flanders drew special attention to measles and stressed that measles vaccination is free of charge for adults up to 45 years of age [9]. In Wallonia and Brussels, special attention was given to the measles elimination target and the need for high coverage with two doses of the MMR vaccine. In Brussels, the information campaign also underlined the importance to vaccinate young adults [10].

Different other control measures were taken in relation to the outbreaks. In a hospital, it was difficult to find the source of infection and the non-immunised exposed staff was screened by laboratory investigation (IgM, IgG and PCR). In a region where many nosocomial transmissions occurred, the regional health authorities visited the local hospitals and gave advice on control measures. This resulted in increased awareness among the staff, a larger number of staff being vaccinated, better triage in the emergency department and better isolation measures. Finally, a small outbreak in an asylum centre in Wallonia was controlled by timely vaccination of 300 persons in the centre.

## Discussion

The analysis of these different outbreaks shows once again that measles is difficult to eliminate as targeted by the WHO’s measles elimination plan [11]. In Belgium, the measles vaccine was available on the market in 1974 [12]. Vaccination with measles-mumps-rubella (MMR) combined vaccine was introduced free of charge in the routine vaccination programme in Belgium in 1985 (one dose) and 1995 (two doses). No catch-up campaign for those born before 1985 or before 1974 took place. The vaccination coverage for the first dose of the MMR vaccine was 94.1% in the Brussels Capital Region (2012), 96.6% in Flanders (2012) and 95.6% in Wallonia (2015) [13-15]. In 2012, coverage for the second dose of MMR was 92.5% in Flanders [15]. For Wallonia and the Brussels Capital Region, a new survey on vaccination coverage data for the second dose of MMR is ongoing in 2016; the latest data available are from 2008–09, showing 75.5% in Wallonia and 75.5% in the Brussels Capital Region [16]. The second MMR dose is systematically offered at school in all three regions. Differences between regions exist, but comparison is difficult because of the different survey periods. 

In Belgium, the first dose of MMR is given at the age of 12 months and the second dose of MMR is given at the age of 10 to 13 years [2]. The WHO advocates giving the second dose one month after the first one [11]. In Belgium, the timing for the second dose is historical and linked to the rubella/mumps vaccine for which the programme already existed and was well incorporated in the routine vaccination schedule [17].

The biggest challenges encountered during these measles outbreaks in Belgium were severe complications, mainly in adults, and nosocomial transmissions. Known complications of measles are otitis media, pneumonia, and encephalitis. Rare complications observed during these outbreaks included rhabdomyolysis and hepatic cytolysis, known rare complications of measles [18,19]. Nosocomial transmission is an important mode of measles transmission in low incidence countries [20,21].

The lessons learnt from these outbreaks pertain to four levels. Firstly, the level of the patients: more than half of the cases (37/67) were unvaccinated and almost a third (20/67) did not know their vaccination status. We did not find distrust or anti-vaccine beliefs to be an important factor for not being vaccinated. Unintentional behaviour of some patients augmented the number of nosocomial infections. Some of them went directly to a crowded emergency department without consulting a general practitioner. Secondly, doctors have an important role in early recognition and diagnosis of measles. However, sometimes lack of familiarity with measles or cases with atypical symptoms lead to a late diagnosis or referral to emergency services and more secondary cases [21]. We noticed that some healthcare workers considered measles as a harmless disease. Moreover, some cases were notified late or only detected during contact tracing. Thirdly, better organisation at hospital level can improve the control of an outbreak. In some hospitals visited, there was no efficient triage in the (often overcrowded) waiting rooms of the emergency department. In other departments, there were isolation measures, but these seemed not sufficient to prevent further spread of measles. The triage in the emergency department could be improved by education of medical staff in early recognition of highly contagious diseases. Most of the hospitals visited did not have a specific procedure for measles cases. These are tasks of the hygiene department of the hospital. The department of occupational medicine also plays an important role in the control of outbreaks and in verifying if healthcare workers are adequately protected against measles. The number of staff involved in the current outbreaks in Belgium was rather small. However, non-immunised healthcare workers are at increased risk of contracting and spreading measles and therefore checking their immune status remains important to prevent the further propagation of nosocomial infections [20]. The fourth level in measles outbreaks are the public health authorities. They have an important role in contact tracing and taking control measures, which can be very resource-intensive.

## Conclusion

The measles outbreaks described here highlight the rapid propagation of measles by nosocomial transmission and the possibility of severe measles complications in adults. To achieve measles elimination, besides strengthening surveillance and improving vaccination coverage in the general population, immunisation strategies should be directed at healthcare workers and those working with children too young to be vaccinated.
